# Effect of normal load and roughness on the nanoscale friction coefficient in the elastic and plastic contact regime

**DOI:** 10.3762/bjnano.4.7

**Published:** 2013-01-28

**Authors:** Aditya Kumar, Thorsten Staedler, Xin Jiang

**Affiliations:** 1 Institute of Materials Engineering, University of Siegen, Paul-Bonatz-Str. 9-11, 57076 Siegen, Germany

**Keywords:** nanoindentation, nanotribology, scratch testing, surface roughness

## Abstract

The influence of applied normal load and roughness on the tribological behavior between the indenter and sample surface during nanoindentation-based scratching has been experimentally investigated by using different surfaces (fused silica and diamond-like carbon) featuring various degrees of roughness. At a sufficiently low applied normal load, wherein the contact is elastic, the friction coefficient is constant. However, at increased normal loads the contact involves plastic deformation and the friction coefficient increases with increasing normal load. The critical load range for a transition from predominantly elastic to plastic contact, between the indenter and sample surface, increases with increasing size of indenter and decreases with roughness. Distinct differences between the present experimental results and the existing theoretical models/predictions are discussed.

## Introduction

Understanding the contact phenomena underlying tribological processes is fundamental to many basic and applied problems, such as wetting, capillarity, adhesion, lubrication, sealing, hardness, micro/nanoindentation, atomic-scale probing, surface modification and manipulation [1–3]. The contact of two bodies may be defined by the influential parameters such as the applied load or contact force between the contacting bodies, real contact area, real contact pressure and its distribution over the contacting surface, and actual separation between both bodies. Engineered surfaces are not perfectly smooth and possess finite roughness. Many of the existing models of rough surface topography are based on the relative distribution of asperities within the contact. In order to understand the effect of roughness, statistical rough surface contact modes have been introduced starting from the very early work of Abbot and Firestone in 1933 [4] for purely plastic contact and the classical work of Greenwood and Williamson in 1966 (GW model [5–6]) for purely elastic contact. According to the GW model, the establishment of elastic or plastic contact is independent of the applied normal load and only influenced by the physical properties of the contacting bodies. To account for elastic–plastic asperity contacts, Chang (CEB model [7–8]) extended the GW model to an elastic–plastic regime assuming the volume conservation law for asperities. However, the CEB model neglects the higher plasticity of the contact in resistance to the additional tangential loading. Later, Kogut and Etsion (KE model [9]) improved the CEB model by accounting for the resistance to sliding of plastically deformed asperities using the finite element method. According to them, the contact parameters, such as separation, real area of contact, and real contact pressure, are functions of the plasticity index and contact load. Their recent work [10] showed that the static friction coefficient (ratio of friction force and normal load) depends on the external force and nominal contact area. Recently, FEM based work by Flores et al. [11] showed that the apparent friction coefficient at a low level of normal load, featuring a predominantly elastic contact, is constant; and at a high level of load, featuring a predominantly plastic contact, is increased. However, this model underestimates the apparent friction coefficient, especially for the ultralow load regime, as the apparent friction coefficient decreases here with increasing load following Hertzian behavior. Another study [12] shows the effect of normal load on the friction coefficient. In this work the friction coefficient is defined as the slope of the friction force with respect to normal load [13]; it is observed that the coefficient of friction in the low load region of elastic deformation is less than that detected in the high load region of plastic deformation. Despite being based on physical and chemical principles as well as the huge amount of experimental work that has been carried out, up to now no complete understanding of the behavior of the friction force or friction coefficient with respect to the contact regime has been achieved, i.e., the effect of load and/or roughness on the friction coefficient is not fully understood for different contact modes. Today the technological progress in scanning-probe techniques opens up the potential to study contact phenomena on the single-asperity level [14]. Here scanning nanoindentation in particular allows for quantitative assessment of the forces involved.

In this paper, various scratch tests with different linearly increasing normal loads for surfaces featuring different roughness values (fused silica (FS) and diamond-like carbon (DLC)) have been carried out. Aside from the normal load, the tip radius of the conical diamond indenter has been varied in these experiments. The friction coefficients were measured and compared to the GW and the KE model as well as the FEM-based model mentioned above. The goal was to study the effect of the applied normal load and roughness on the friction coefficient and the critical normal load regime for a transition from a predominantly elastic to a plastic contact between the indenter and surface of the sample during a nanoindentation-based scratch test with linearly increasing load.

## Experimental

**Samples:** As mentioned above, fused silica and DLC were chosen as sample materials. The fused silica was provided as a standard sample by Hysitron Inc. The DLC samples, 1µm thick films on Si(100) wafer, were synthesized by chemical vapor deposition (Balzer BAS 450) utilizing a gas mixture of argon and acetylene at a bias voltage of −950 and −350 V, respectively.

**Topographical characterization:** The surface morphology was characterized by atomic force microscopy (AFM, Park Systems Corp. XE-100). Noncontact AFM was used to obtain detailed information about surface topography and surface roughness. The samples were imaged with commercial tips featuring a nominal tip radius of 10 nm in a feedback-controlled mode on all three axes. Five 8 × 8 μm^2^ images with a pixel resolution of 512 × 512 were taken at different surface positions on each sample in order to derive the corresponding RMS roughness. The appropriate topography of the conical indenters utilized in the present work was also characterized. The resulting roughness of the 1 µm conical indenter was found to be negligible. The 20 µm conical indenter featured topography aside from the overall macroscopic conical one (with spherical end cap). However, its characteristic length scale was significantly larger than that of the samples studied here. For these reasons, we refrained from taking the indenter roughness into account in both cases.

**Figure 1:**
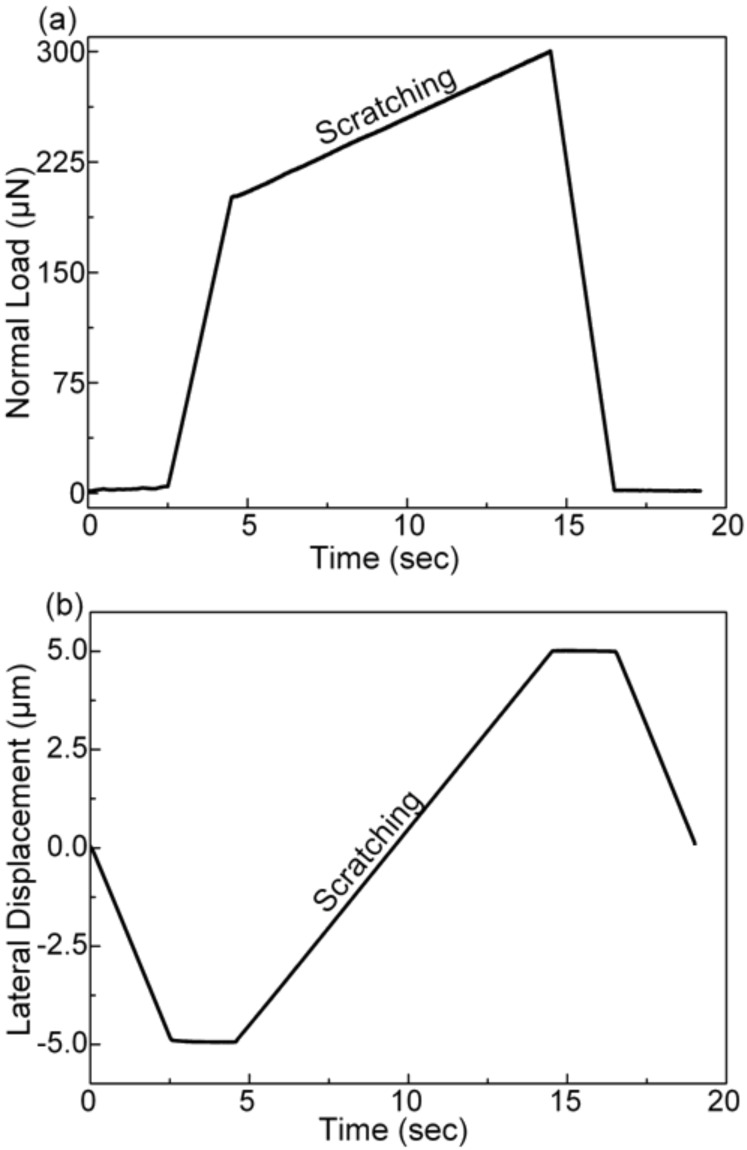
Example for a load (a) and displacement scheme (b) of one of the individual scratch segments used in this work.

**Mechanical and tribological characterization:** The mechanical and tribological sample characterization was carried out by a transducer-based scanning nanoindenter (TriboIndenter, Hysitron Inc.) in a laboratory environment (RT and 50% RH). The mechanical properties of the samples were evaluated with a Berkovich diamond tip following the procedure proposed by Oliver and Pharr [15–16]. The samples were probed at three different spots. At each spot 25 indents were placed in a grid pattern (5 × 5 indents with 20 µm spacing) varying in final load from 10 mN to 200 µN (100 µN/s loading and unloading rate, 5 s hold time at maximum load). Preceding the mechanical analysis, tribological tests were carried out with two conical diamond tips featuring nominal tip radii of 1 µm and 20 µm (90° cone opening angle). The corresponding real tip radii, determined by fitting of a Hertzian contact to low-load indents into fused silica, are 0.7 µm and 4.5 µm, respectively. Later on these real radii will be used in the context of all calculations. As preliminary testing confirmed that results are not influenced by the fashion of load ramping, i.e., increasing or decreasing load during scratching, only unidirectional scratch tests with linearly increasing load were performed. One load and lateral displacement scheme of a scratch segment, which will be described below, is shown in Figure 1. For all scratch tests in the work presented here the scratch speed, minimum distance between two scratches, and number of scratches for a particular load were set to 1 µm/sec, 20 μm, and 10, respectively.

**Figure 2:**
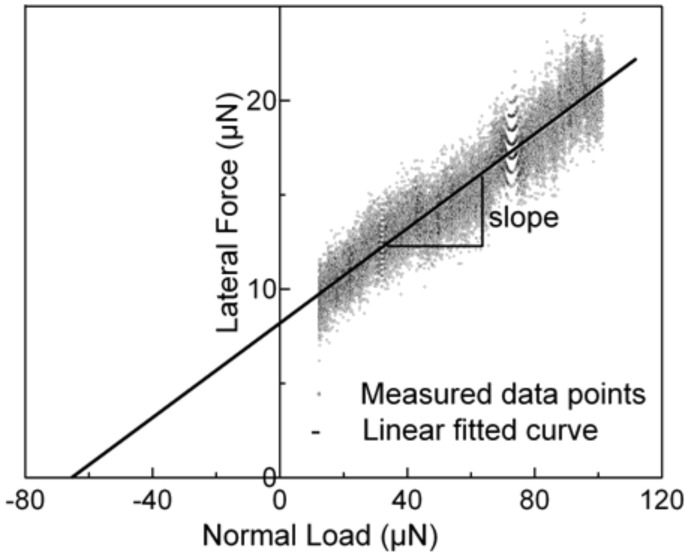
Lateral force versus normal load plot for the fused silica sample in contact with the 1 µm conical indenter. Friction coefficient is estimated by a linear fitting routine. See text for details.

In order to identify the suitable normal-load range, i.e., the range that did not feature any artifacts that may be dominated by instrumental boundary conditions, the total normal-load range of each scratch was divided into segments. For practical reasons these segments had to be small but at the same time had to contain an adequate number of data points to be analyzed. In this present case the segment size ranged from 300 µN to 20 µN depending on sample roughness and indenter radius. Here the 300 µN segment size corresponds to a scratch test of the smooth fused silica sample carried out with the large conical indenter, and the 20 µN segment size corresponds to a test of the rough DLC sample utilizing the small conical indenter. The suitable load range was then defined as the range from the minimum normal load of 10 µN up to either the maximum normal load of the instrument, i.e., 10 mN, or the first segment that featured a maximum lateral-load difference larger than its segment size. The latter case usually can be attributed to some stick–slip event, which will contain a strong influence of the properties of the transducers spring setup. Therefore, such segments in the present work will not be considered. Once the normal load range was established, the slope of a linear trend line fit to each segment was taken as the coefficient of friction of the segment at a normal load equal to the center of the segments. This procedure ensures the elimination of any nonzero measured friction force that may be present at a normal load of zero, see Figure 2. This is usually explained by an additional load term due to an intrinsic adhesive force and/or artifacts generated by the equipment. The adhesion force term itself consists of various attractive forces such as capillary, electrostatic, van der Waals, and others.

## Results

**Figure 3:**
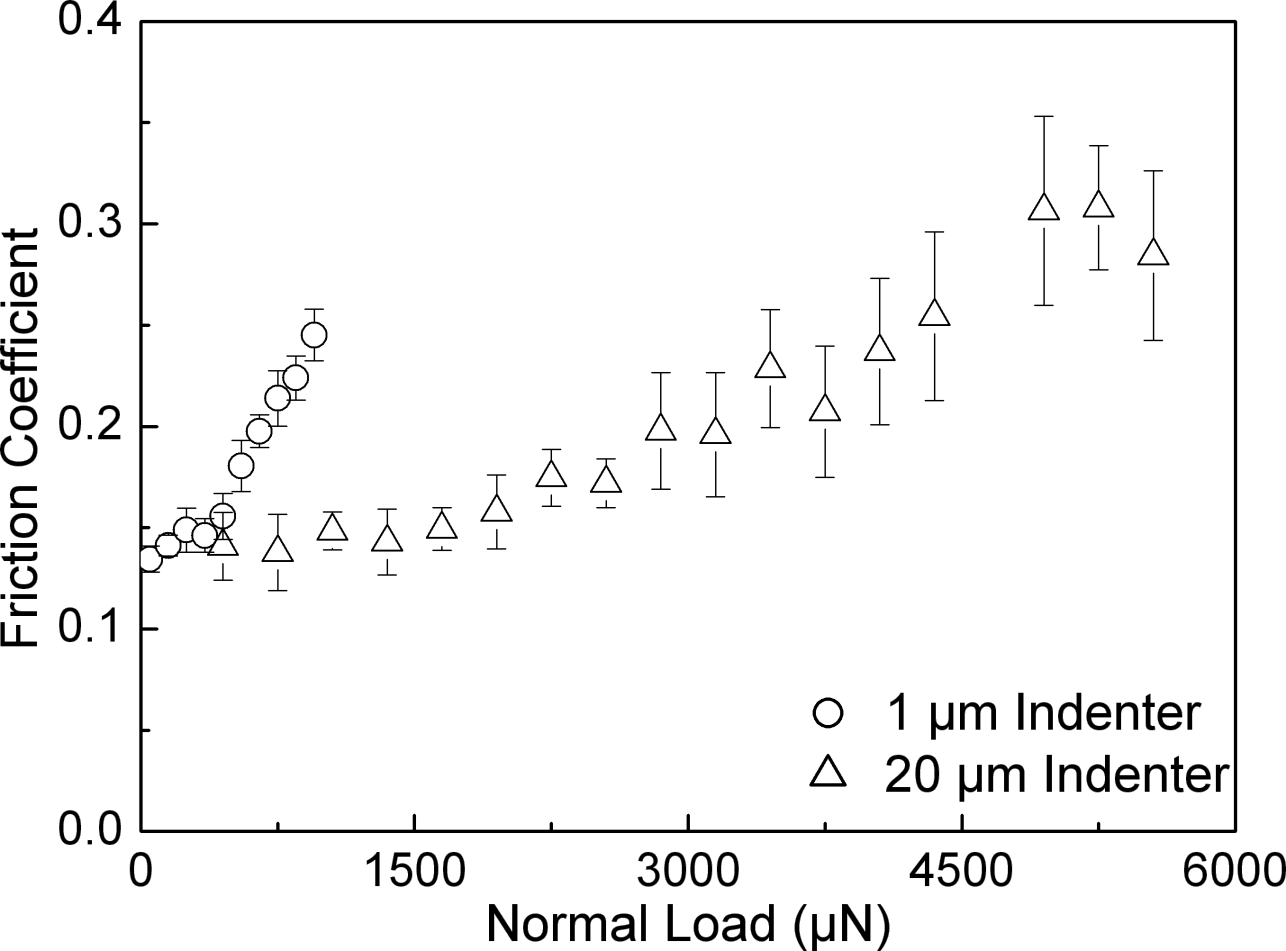
Friction coefficient versus normal load for the fused silica sample derived with both conical indenters. The error bars show the standard deviation of data.

**Figure 4:**
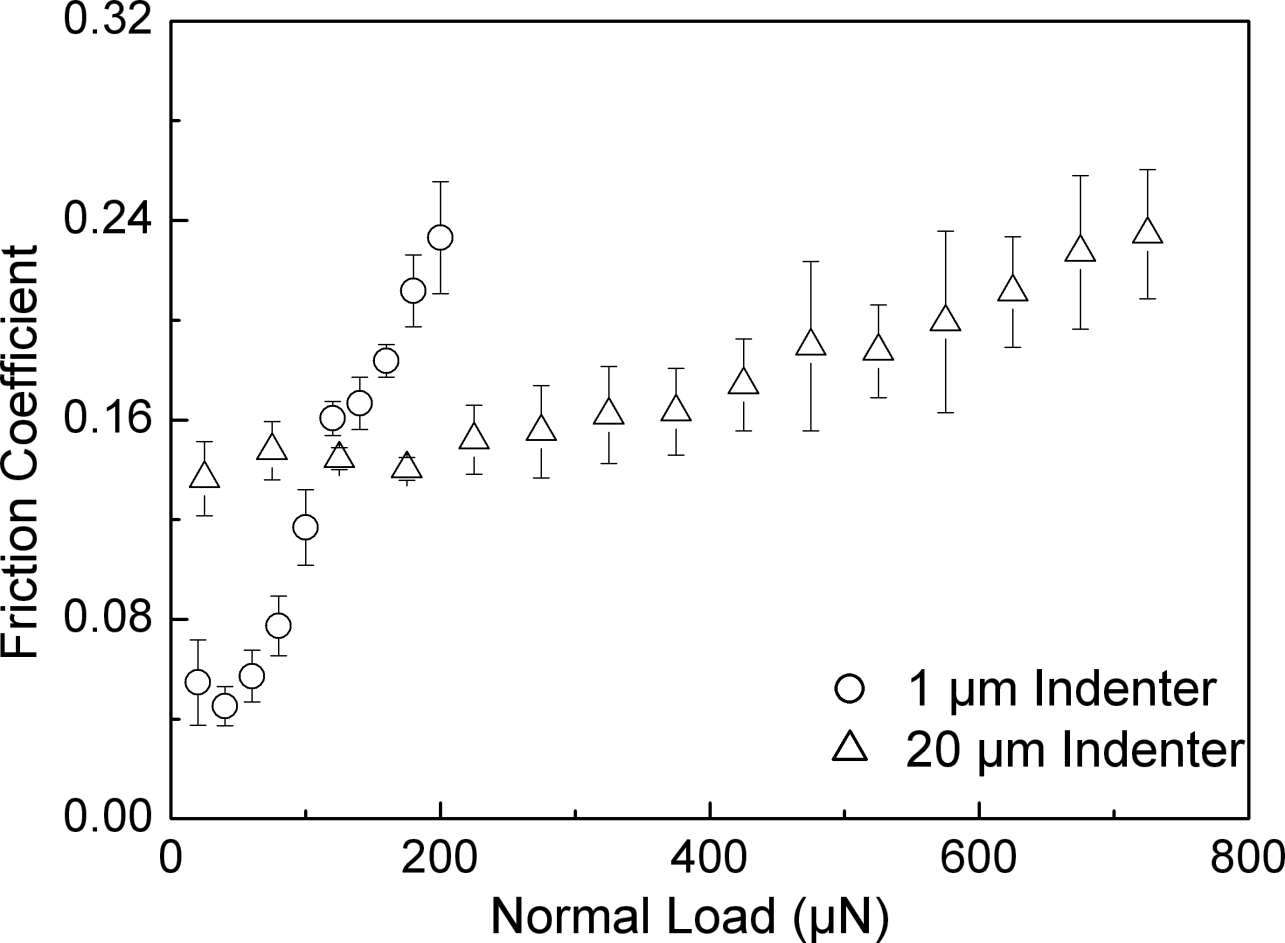
Friction coefficient versus normal load for the smooth DLC sample derived for both conical indenters. The error bars show the standard deviation of the data.

**Figure 5:**
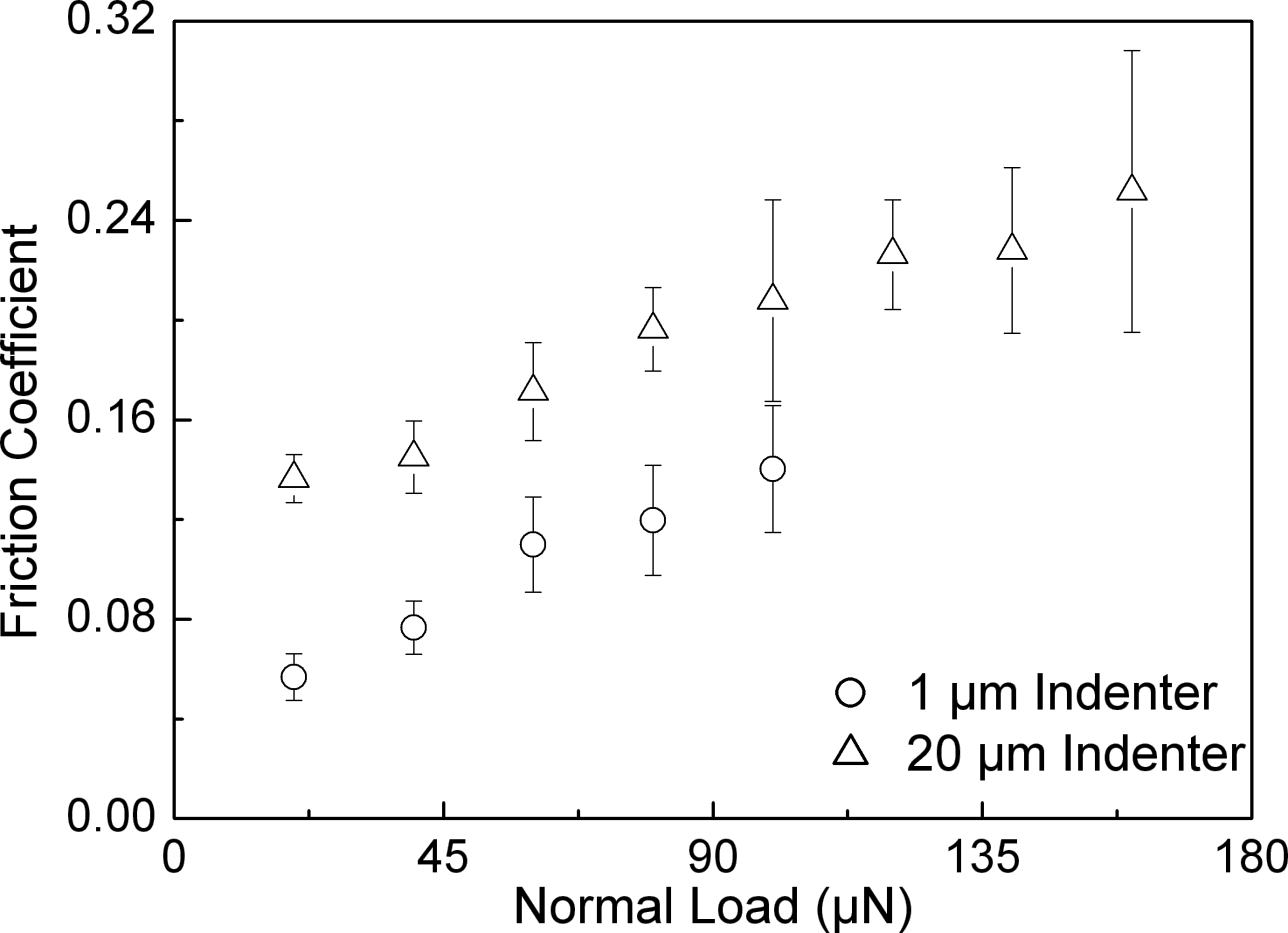
Friction coefficient versus normal load for the rough DLC sample derived for both conical indenters. The error bars show the standard deviation of the data.

The roughnesses along with the mechanical properties of the samples are given in Table 1. Analyzing the tribological data, some distinct differences in the behavior of the three samples that are the subject of this work are revealed. In order to take a detailed look at the behavior of the friction coefficient with respect to the applied normal load, the corresponding results have been plotted for all three samples and are shown in Figure 3, Figure 4, and Figure 5. These figures also show the error bars that were measured from multiple test data. The friction coefficient for all three samples was always higher for the 20 µm radius conical indenter than for the 1 µm radius conical indenter. Generally a low friction coefficient is observed at an early stage of each scratch, i.e., low applied normal loads. An increase of the normal load during scratching typically results in an increased coefficient of friction. This increase can be either continuous as in case of the rough DLC sample, see Figure 5, or the increase is found only if the normal load exceeds some certain critical load. The actual critical load of a transition from a low, apparently constant coefficient of friction to the linearly increasing one depends on the sample material and the roughness as well as the indenter used.

**Table 1:** Topographical and mechanical properties of the fused silica, the smooth, and the rough DLC sample.

sample	hardness *H* (GPa)	reduced Young’s modulus E* (GPa)	roughness σ_s_ (nm)	curvature constant *k*_s_ × 10^−3^ (nm^−1^)	hardness coefficient *K*

FS	9.21 ± 0.29	69.55 ± 1.15	0.62 ± 0.02	2.93 ± 0.35	0.5237^a^
smooth DLC	21.44 ± 3.01	169.39 ± 11.82	4.05 ± 0.06	6.46 ± 0.21	0.577^b^
rough DLC	23.27 ± 5.58	187.26 ± 26.23	11.69 ± 0.66	4.06 ± 0.58	0.577^b^

^a^Poisson ratio ν = 0.17; ^b^ν = 0.30

It is generally accepted that these transitions correspond to a transition from a predominantly elastic to a predominantly plastic contact between the sample and the indenter. For this reason it is obviously not possible to provide precise normal-load numbers for such a transition, as the contact between two rough surfaces will typically feature asperities that are deformed elastically along with those that are already plastically deformed. Table 2 (see below) gives the appropriate ranges of normal loads during scratching for which the above-mentioned transitions have been observed in the experiments carried out here. Silica as well as the smooth DLC sample shows a transition between a predominantly elastic and a predominantly plastic contact. The observed values here are 400–500 µN and 1800–2100 µN as well as 50–70 µN and 200–250 µN for tests carried out with the 1 µm and the 20 µm conical diamond indenter on silica (Figure 3) as well as on the smooth DLC sample (Figure 4), respectively. For the rough DLC sample any load regimes featuring a predominantly elastic contact were not identified. Therefore, no transition was observed and a predominantly plastic contact is established already at very low loads (Figure 5). It was also observed that the critical load range increases with increasing indenter size. These findings show that the combination of mechanical properties, sample roughness, and indenter radius are key parameters in determining the contact characteristics, i.e., whether the indenter is in a predominantly elastic or plastic contact, at a given normal load.

In order to take a closer look at the influence of roughness on the contact characteristics we calculated various plasticity indices that have been proposed in the literature. The first here is the one given by the GW model [5–6], (E^*^/*H*)(σ_s_*k*_s_)^1/2^, where *H* is the hardness, E^*^ is the reduced Young’s modulus, σ_s_ is the surface roughness, and *k*_s_ is the curvature constant. In the case of the fused silica it was found to be less than unity, whereas both DLC samples feature plasticity indices greater than unity (Table 2). Another modified plasticity index given by the KE model [9] was calculated as (2E^*^/π*KH*)(σ_s_*k*_s_)^1/2^, where *K* represents the hardness coefficient (*K* = 0.454 + 0.41ν) and ν is the Poisson ratio. In the present case, following these calculations, the plasticity index of 0.39 for silica (ν = 0.17) and plasticity indices of 1.41 and 1.93 for smooth and rough DLC (ν = 0.30), respectively, were obtained as shown in Table 2. The FEM-based work presented by Flores et al. [11] provides critical loads for a predominantly elastic (normal load < 25ε^2^*R*^2^σ) and predominantly plastic contact (normal load > 225ε^2^*R*^2^σ), where ε is yield strain, σ is yield strength, and *R* is the indenter radius. At intermediate normal loads (25ε^2^*R*^2^σ to 225ε^2^*R*^2^σ) the contact characteristic is a mixture between the two. These critical values for all three samples and both indenters are calculated. The results are also shown in Table 2. Although, the general trend of the friction coefficient with increasing normal load is experimentally verified, i.e., initial constant low value followed by a linearly increasing coefficient of friction after a critical normal-load range has been exceeded, the absolute values of the calculated and experimentally found load boundaries differ significantly. The most striking differences in this context are the load boundaries in the case of the smooth and rough DLC samples. The calculations lead to very similar boundaries for both DLC samples, whereas the experimental tests show huge differences between the two. Here the smooth DLC sample showed a predominantly plastic contact regime. The rough DLC sample on the other hand featured no such regime in the normal-load range tested in this work.

**Table 2:** Plasticity indices and critical load ranges for the fused silica, the smooth DLC, and the rough DLC sample.

sample	plasticity index^a^	plasticity index^b^	1 µm indenter		20 µm indenter		approximated critical load range (µN)	

			25ε^2^*R*^2^σ (µN)	225ε^2^*R*^2^σ (µN)	25ε^2^*R*^2^σ (µN)	225ε^2^*R*^2^σ (µN)	1 µm indenter	20 µm indenter

FS	0.32 ± 0.02	0.39 ± 0.02	68.55	616.95	2832.98	25496.77	400–500	1800–2100
smooth DLC	1.28 ± 0.03	1.41 ± 0.03	136.76	1230.83	5651.77	50965.98	50–70	200–250
rough DLC	1.75 ± 0.13	1.93 ± 0.14	136.66	1238.95	5647.68	51201.50	—	—

^a^Greenwood and Williamson model; ^b^Kogut and Etsion model.

## Discussion

In this work the tribological contact behaviors between two conical diamond indenters and fused silica as well as diamond-like carbon samples featuring different roughness during nanoindentation-based scratch test carried out with linearly increasing normal load were investigated. The friction coefficients were segmentally calculated from the slope of a linear fit to the lateral force versus normal load. The friction coefficient is found to increase with the size of indenter due to obvious reasons of increasing contact area, and hence the critical load regime will change accordingly. The results were compared with predictions by the GW as well as the KE model. In both cases the models estimate a predominantly elastic contact for the tests on fused silica and predominantly plastic contact for both DLC samples. This could not be verified by experiments as the fused silica sample showed a transition from predominantly elastic to plastic contact and the smooth DLC sample featured a predominantly elastic contact regime. Therefore, both observations are in contradiction to the models.

The general trend of a transition from a predominantly elastic contact regime featuring a low constant friction coefficient, to a predominantly plastic contact characterized by an increasing friction coefficient with increasing load, suggested by FEM calculations of Flores et al., was experimentally reproduced. However, the load boundaries predicted by the FEM model significantly overestimate the ones that were experimentally found. In addition to that, the FEM model fails to reproduce the significant differences between the two DLC samples of different roughness.

In summary the results presented here show that to date the existing contact models are not able to simulate the behavior of the friction coefficient during nanoindentation-based scratch tests. Especially the influence of sample roughness is not well understood. Therefore, the authors are in the process of carrying out a series of systematic tests on various samples featuring roughness variations allowing for a more detailed analysis of the effect of roughness on the load dependence of the coefficient of friction. The findings will be the content of a future publication.

## Conclusion

In this work, the influence of the applied normal load and roughness on the tribological behavior between the indenter and sample surface using a nanoindenter has been studied. The transition from a predominantly elastic contact regime featuring a constant coefficient of friction to a predominantly plastic contact characterized by an increasing coefficient of friction with increasing load was experimentally observed. It was found that the critical load range for a transition from predominantly elastic to plastic contact increases with increasing size of indenter and decreases with surface roughness. The experimental results were compared with the predictions of the model by Greenwood and Williamson and the one by Kogut and Etsion, as well as the FEM-based model by Flores et al. None of the presently available theoretical models was able to quantitatively describe the experimental results.
